# Supplementary material for: High-Performance Anion Exchange Chromatography with Pulsed Amperometric Detection (HPAEC–PAD) and Chemometrics for Geographical and Floral Authentication of Honeys from Southern Italy (Calabria region)
Source: Foods. 2020 Nov 7;9(11):1625. doi: 10.3390/foods9111625 (PMC7694965; doi:10.3390/foods9111625)
Supplement: Supplementary file 1 [file foods-09-01625-s001.pdf]

Table S1 Confusion matrix for Calabrian honey classify based on botanical origin

	Acacia	Chestnut	Citrus	Eucalyptus	Sulla	Tot.	*% Discrimination index*
Acacia	10	0	0	0	0	10	100.00%
Chestnut	0	16	0	0	0	16	100.00%
Citrus	0	0	12	0	1	13	92.31%
Eucalyptus	0	0	1	9	0	10	90.00%
Sulla	0	0	1	0	11	12	91.67%
Tot.	10	16	14	9	12	61	95.08%

Table S2 Confusion matrix for Acacia samples

From \ To	Acacia Calabria	Acacia China	Acacia Europe	Tot	% exact
Acacia Calabria	10	0	0	10	100.00%
Acacia China	0	6	0	6	100.00%
Acacia Europe	0	0	7	7	100.00%
Tot.	10	6	7	23	100.00%
